# Identifying Source Populations and Genetic Structure for Savannah Elephants in Human-Dominated Landscapes and Protected Areas in the Kenya-Tanzania Borderlands

**DOI:** 10.1371/journal.pone.0052288

**Published:** 2012-12-26

**Authors:** Marissa A. Ahlering, Lori S. Eggert, David Western, Anna Estes, Linus Munishi, Robert Fleischer, Melissa Roberts, Jesus E. Maldonado

**Affiliations:** 1 Division of Biological Sciences, University of Missouri, Columbia, Missouri, United States of America; 2 Center for Conservation and Evolutionary Genetics, Smithsonian Conservation Biology Institute, National Zoological Park, Washington, District of Colombia, United States of America; 3 African Conservation Centre, Nairobi, Kenya; 4 Department of Environmental Sciences, University of Virginia, Charlottesville, Virginia, United States of America; 5 Wildlife Conservation Society, Tarangire Elephant Project, Arusha, Tanzania; 6 Department of Vertebrate Zoology, National Museum of Natural History, Smithsonian Institution, Washington District of Colombia, United States of America; Texas A&M University, United States of America

## Abstract

We investigated the genetic metapopulation structure of elephants across the trans Rift Valley region of Kenya and Tanzania, one of the remaining strongholds for savannah elephants (*Loxodonata africana*) in East Africa, using microsatellite and mitochondrial DNA (mtDNA) markers. We then examined this population structure to determine the source population for a recent colonization event of savannah elephants on community-owned land within the trans rift valley region. Four of the five sampled populations showed significant genetic differentiation (p<0.05) as measured with both mtDNA haplotypes and microsatellites. Only the samples from the adjacent Maasai Mara and Serengeti ecosystems showed no significant differentiation. A phylogenetic neighbour-joining tree constructed from mtDNA haplotypes detected four clades. Clade four corresponds to the F clade of previous mtDNA studies that reported to have originated in forest elephants (*Loxodonta cyclotis*) but to also be present in some savannah elephant populations. The split between clade four and the other three clades corresponded strongly to the geographic distribution of mtDNA haplotypes across the rift valley in the study area. Clade four was the dominant clade detected on the west side of the rift valley with rare occurrences on the east side. Finally, the strong patterns of population differentiation clearly indicated that the recent colonists to the community-owned land in Kenya came from the west side of the rift valley. Our results indicate strong female philopatry within the isolated populations of the trans rift valley region, with gene flow primarily mediated via male movements. The recent colonization event from Maasai Mara or Serengeti suggests there is hope for maintaining connectivity and population viability outside formal protected areas in the region.

## Introduction

African savannah elephants (*Loxodonta africana*) once ranged across much of the African continent [1], [2], but the increase in human population, and resulting habitat loss, fragmentation and continuous poaching for ivory, have resulted in dramatic population declines and isolation. Populations in Kenya were particularly hard hit by illegal killing in the 1970s and 1980s prior to the ivory ban [3], [4], causing extreme fragmentation and isolation of elephant populations as they retreated into protected areas. This anthropogenic isolation has created a metapopulation structure for elephants in Kenya and Tanzania. Recently, however, elephant numbers in protected areas have begun to rise [5], increasing the potential for elephants to expand their movements into more human-dominated landscapes. In southern Kenya, elephants have begun recolonizing community-owned conservation areas, and understanding where these elephants came from will help identify movement corridors for conservation and management purposes in the region.

Genetic information is often used to detect population structure, estimate dispersal, and identify sources for a recently established population [6]. To use genetic information to identify source populations of recent colonists, information about the surrounding populations and the genetic structure of the regional metapopulation is necessary. Population structure has been examined at the continental [7], [8], regional [2], and even country level [4], [9] for savannah elephants. Across eastern and southern Africa, savannah elephants show population structure at the largest scale and patterns of isolation by distance at a regional level, with evidence of long-term gene flow still apparent [2]. On a more local scale for many species, population structure is often apparent using mitochondrial DNA (mtDNA) with more subtle structure detected using nuclear DNA [4], a pattern which is often explained by isolated populations showing female philopatry and male-biased dispersal [9]. In species, such as elephants, where female philopatry is so strong that females rarely disperse, mtDNA and biparentally inherited markers often show incongruent patterns [10]–[18]. However, in this case, a large number of female savannah elephants have dispersed across the landscape, and the strong female philopatry isolating mtDNA should be useful in helping to determine the population origin for these elephants.

Evaluating localized genetic metapopulation structure and sources for recent colonization events can elucidate movement patterns. To maintain viable populations, elephants need areas larger than most protected areas can provide [19], and identifying elephant movement patterns can accelerate land use planning decisions for habitat protection that are important for keeping people and elephants safe. In this study, we evaluate the genetic metapopulation structure of elephants across the rift valley region that spans the Kenya-Tanzania border. This region encompasses some of the largest and most visited populations of elephants in East Africa [5], and therefore, an understanding of metapopulation structure is important for conservation of elephants and for the vibrant wildlife tourism industry in the borderlands region. We then use this metapopulation information to identify a source for a recently established elephant population on community-owned land in the rift valley. The recently established population of interest for this study was located on two adjacent Maasai community group ranches in southern Kenya, Olkiramatian and Shompole. In 2000, these communities each established adjacent Community Conservation Areas (CCAs) setting aside 20,000 ha of contiguous habitat for wildlife conservation and ecotourism and for use as a drought refuge for livestock. Shortly after establishment, elephants began recolonising the CCA after an absence of at least two decades (Western pers. obs.). Here we determine the patterns of metapopulation genetic structure in the region and use this information to identify the source population of the elephants now occupying the CCA.

## Methods

### Study Area

**Figure 1:**
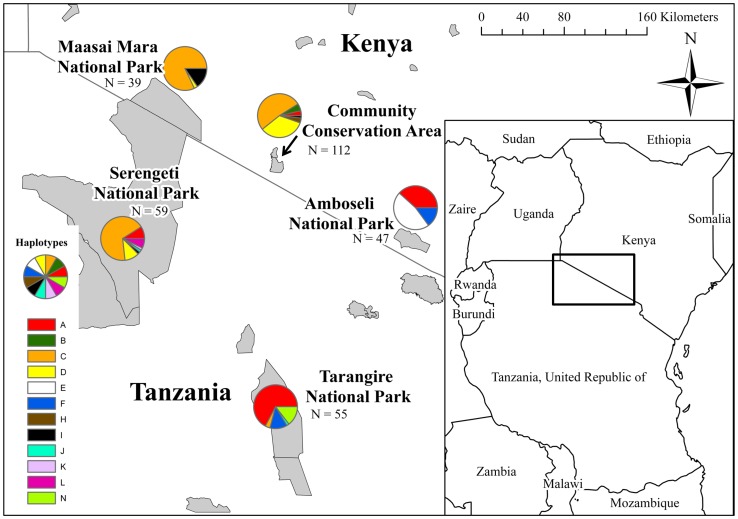
Map of Sampling Locations. Map of the sampling locations in southern Kenya and northern Tanzania, including the sample size of individuals and mtDNA haplotype frequencies for each site; the Rift Valley splits the study area, Maasai Mara and Serengeti are on the west side of the valley, Amboseli and Tarangire are on the east side of the valley, while the CCA is located within the Rift Valley.

Olkiramatian and Shompole (Fig. 1, 2°00′126″S, 35°59′331″E) are located in the Rift Valley province of Kenya. The dominant land use for both group ranches is migratory livestock grazing by cattle, sheep, and goats. A small agricultural settlement is confined to the riverine areas along the base of the Nguruman Escarpment. The primary habitat on both group ranches is open savannah transitioning to acacia woodland near the rivers and rift valley escarpment. Permission was obtained from both communities to collect samples on their land. With permission from the Kenya Wildlife Service, we sampled the two primary surrounding populations of elephants in southern Kenya, Amboseli National Park and Maasai Mara National Game Reserve. We also sampled the two major elephant populations across the border in northern Tanzania in Serengeti National Park and Tarangire National Park with the consent of the Tanzania Wildlife Research Institute. The CCA lies within the Rift Valley, which splits the study area. Maasai Mara and Serengeti are located on the west side of the Rift Valley while Amboseli and Tarangire are located on the east side of the Rift Valley.

### Field Methods

We collected fresh dung samples non-invasively from which we obtained DNA for genetic analyses. Because we worked only with faecal samples and did not interact with the animals, animal care and use permits were not required. Within the CCA, we collected faecal samples from as many of the individuals as possible. In the parks, we attempted to get a representation of elephants from across the parks by collecting fresh dung from different parts of the parks over a short time frame, trying not to sample closely related individuals. Samples were only collected from fresh dung, estimated to be less than 12 h old.

From each dung pile, we collected material from the external region of one dung bolus for DNA analyses. The sample vials were boiled in water prior to adding the buffer for 15 min to destroy pathogens and preserved with Queens College buffer (20% DMSO, 0.25 M EDTA, 100 mM Tris, pH 7.5, saturated with NaCl) [20]. We recorded GPS coordinates for all dung samples.

In the CCA, we collected dung samples between May and July in 2007, and in 2008 we collected samples between February and April. Samples from Amboseli and Maasai Mara were collected in June 2007. Samples from Serengeti were collected in August 2009, and samples from Tarangire were collected in November 2009. Dung samples were imported into the US for DNA analysis (Kenya samples USDA permit #48529, Tanzania samples USDA permit #104141).

### Genetic Methods

DNA was initially extracted following the methods of Eggert et al. [21]. Samples that were re-extracted for non-invasive replication followed the modified protocols of Archie et al. [22] for the QIAamp DNA Stool Mini Kit (Qiagen, Valencia, CA). For mtDNA analyses, a 650 bp fragment of the control region was PCR amplified and Sanger sequenced for all DNA samples (MDL3 & MDL5) [23]. To identify individuals and examine relatedness, we genotyped all samples at 12 microsatellite loci (FH60R, FH94R, FH48R, FH67, FH126, LA4, LA5, LA6R, LafMS02, LaT05, LaT08, LaT13R) [22], [24], [25]–[28]. To increase our PCR amplification success, we redesigned the forward primer for LaT13 from Archie et al. [22] to shorten the fragment length from 234–262 bp to 177–217 bp (5′-3′ F-GCCAGTGTCATGAACAGCAT).

We performed PCR in 25 µl volumes containing 0.5 U Ampli*Taq* Gold DNA Polymerase (Applied Biosystems, Foster City, CA), 1X PCR Gold Buffer, 0.2 mM each dNTP, 0.4 µM fluorescently labeled forward primer, 0.4 µM unlabelled reverse primer (both primers unlabelled for mtDNA PCR), 1.5 mM MgCl_2_, 10X BSA (New England Biolabs, Ipswich, MA), and 3 µl of DNA template. PCR profiles began with denaturation at 95°C for 10 min, followed by 40 cycles of 95°C for 30 sec, annealing at locus-specific temperatures (FH48R and MDL3 & MDL5 at 60°C; FH60R, FH94R, FH126, LA6R and LaT05 at 58°C; LaT08 at 55°C; FH67 and LA4 at 54°C; LA5 and LafMS02 at 52°C; LaT13R at 50°C) for 30 sec, and primer extension at 72°C for 30 sec. The final step was one extension cycle at 72°C for 10 min. DNA sequencing and microsatellite fragment analysis of the 2007 samples were conducted at the University of Missouri’s DNA Core Facility using a 3730 DNA Genetic Analyzer (Applied Biosystems), and the sequencing and fragment analysis of the 2008 Kenya samples and all the Tanzania samples were performed on a 3130xl Genetic Analyzer (Applied Biosystems) at the Center for Conservation and Evolutionary Genetics at the Smithsonian Institution in Washington, DC, USA. We ran a set of eight samples on both machines to calibrate internal lane standards and allele calls. DNA from a blood sample of a savannah elephant in the North American captive population was used as a positive control to standardize allele scores, and a negative control was included in all reactions to detect PCR contamination.

Because faecal DNA samples are more prone to error than DNA extracted from blood or tissue samples, we used a multiple tubes approach to perform the genotypying of the samples [29]. DNA from each dung sample was isolated a minimum of two times. Heterozygous genotypes were replicated at least twice and all homozygous genotypes at least five times. After each dung sample was successfully typed at all loci, multilocus genotypes were examined using RelioType [30], a program designed to assess the reliability of an observed multilocus genotype using a maximum-likelihood approach for minimizing genotyping errors. We discarded samples that did not amplify the required two to five times at each locus. To make sure we did not include accidental recaptures in our analyses, we tested the ability of our twelve microsatellites to distinguish between individuals using the probability of identity (*P*_ID_; i.e., the probability of different individuals sharing an identical genotype at random) [31], [32] and the *P*_ID_ between siblings.

We used the Excel Microsatellite Toolkit add-in [33] to identify unique individuals and eliminate recaptures; samples that matched both alleles at all loci were considered recaptures. In the CCA, we also considered pairs of samples that differed by one allele and matched sex to be recaptures due to dropout; this was not an issue for park samples. Recaptures were common in the CCA where we were trying to collect from as many individuals as possible but minimal within the park samples. We determined the sex of the samples as a further check of our recaptures and to help understand potential movement patterns of females (matrilines) revealed by mtDNA. To determine the sex of the Kenyan and Serengeti individuals, we used a molecular sexing method following Munshi-South et al. [34]. After a more efficient sexing protocol was developed, the sex of the individuals from Tarangire was determined following Ahlering et al. [35]. Samples were determined to be males when two fragments of the correct sizes were amplified (representing the two genes: *Zfx* and *Zfy*) and females when a single fragment (representing the *Zfx* gene) was amplified. To eliminate the possibility of misidentifying the sexes due to allelic drop-out, samples sexed as males were confirmed twice, and samples sexed as females were confirmed three times.

### Statistical Analysis

We performed calculations of expected (*H_E_*) and observed heterozygosity (*H_O_*) values and tests for deviations from expectations under Hardy-Weinberg equilibrium and linkage disequilibrium using Genepop 4.0 [36], [37]. In Arlequin v. 3.11 [38], we quantified microsatellite diversity using the number of alleles, the expected and the observed heterozygosity for each population, and we quantified levels of mtDNA variation using indices of nucleotide diversity. We used the program Geneious Pro 5.5.6 [39] to estimate phylogenetic relationships among the mtDNA haplotypes by constructing a neighbor joining tree using the HKY model as indicated by jModelTest 2.02 [40], [41]. To place our haplotypes in a larger phylogenetic framework, we included published sequences from Okello et al. [4], Eggert et al. [7] and Debruyne [17] (Table 1).

**Table 1:** GenBank accession number for haplotypes detected in Kenya and Tanzania from this study and published haplotypes used in the neighbour-joining tree.

Haplotype	GenBank Accession #	Species	Citation
A-F, H-L, N	KC218461–KC218472	Savannah	This study
Addo	AF527682.1	Savannah	Eggert et al. [7]
Angola1	AY741072.1	Savannah	Debruyne [17]
Bia64	AF527679.1	Forest	Eggert et al. [7]
Bmbo37	AF527649.1	Forest	Eggert et al. [7]
Botswana1	AY741074.1	Savannah	Debruyne [17]
DJA39	AF527653.1	Forest	Eggert et al. [7]
Gamba5	KC218473	Forest	This study
K68	AF527643.1	Forest	Eggert et al. [7]
KE2	EU529655.1	Savannah	Okello et al. [4]
KE4	EU529657.1	Savannah	Okello et al. [4]
KE6–10	EU529659.1–EU529663.1	Savannah	Okello et al. [4]
KE14	EU529667.1	Savannah	Okello et al. [4]
KE17–19	EU529670.1–EU529672.1	Savannah	Okello et al. [4]
KE21–22	EU529674.1–EU529673.1	Savannah	Okello et al. [4]
Mozambique1	AY741076.1	Savannah	Debruyne [17]
Namibia2	AY741325.1	Savannah	Debruyne [17]
SamburuA	KC218474	Savannah	This study
SouthAfrica1	AY741320.1	Savannah	Debruyne [17]
Tai29	AF527673.1	Forest	Eggert et al. [7]
Uganda1	AY741323.1	Savannah	Debruyne [17]
Zimbabwe1	AY741075.1	Savannah	Debruyne [17]

To evaluate genetic metapopulation structure and determine the source population for the elephants in the CCA, we used two different statistical approaches. We calculated population pairwise F_ST_ values in Arlequin v. 3.11 [38] for both mtDNA haplotypes (using the Kimura 2-parameter model) and microsatellite allele frequency data. We also used STRUCTURE 2.3.3 [42] to assign the individuals from the CCA to their source population using the microsatellite data. We ran STRUCTURE for values of K from 1 through 9 with 20 replicates for each level of K, a burn-in period of 100,000, and a run length of 1,000,000 under the admixture model and updating for population location. Because we had low F_ST_ values among populations, we used the LOCPRIOR option [43], and we coded the data for five populations: Amboseli, Tarangire, the CCA, Maasai Mara and Serengeti. For comparison, we ran the same STRUCTURE models without any prior information as well as using the POPFLAG option for all populations except the CCA. The Evanno method [44], as visualized using Structure Harvester [45], was used to determine the number of populations (K).

## Results

We identified a total of 312 individuals from the 531 samples collected across all sampled populations in Kenya and Tanzania (Table 2). None of the 12 microsatellite loci showed departure from expectations under Hardy-Weinberg equilibrium or evidence of linkage disequilibrium after Bonferroni corrections. Five of our microsatellite loci can be compared with previously published allele size values for elephants, and the allele sizes for these loci all fall within the size ranges reported for savannah elephants (Table S2). With 12 microsatellite loci we had sufficient power to distinguish individuals and identify recaptures (P(ID)sib = 0.0000624) [32]. All identical genotypes derived from microsatellites also matched in mtDNA haplotype and sex assignment. With our level of replication, RelioType reported the reliability of our genotypes at a probability of 0.99.

**Table 2:** Number of samples collected, successfully genotyped, and individuals for all five populations sampled in Kenya and Tanzania.

Population	Samples Collected	Successfully Genotyped	Total Individuals
Community Conservation Area	317	273	112
Maasai Mara	45	45	39
Amboseli	50	49	47
Serengeti	62	62	59
Tarangire	57	57	55

We detected 12 mtDNA haplotypes across all populations (Row 1,Table 1; Table S1). Mitochondrial DNA nucleotide diversity (Table 3) was highest for Serengeti where we detected seven haplotypes and lowest for Amboseli where we detected only three haplotypes. The microsatellite allelic diversity ranged from 7.25 for both Amboseli and the CCA to 5.75 for Tarangire (Table 4). The observed and expected heterozygosities did not vary much among populations.

**Table 3:** F_st_ value comparison for sampled populations in southern Kenya and northern Tanzania based on microsatellites below the diagonal and mtDNA above the diagonal; percent mtDNA nucleotide diversity for each population is given in bold on the diagonal.

	AM	MM	CCA	SE	TA
AM	**0.2004**	0.7778****	0.4077****	0.4969****	0.1987****
MM	0.0156****	**1.6012**	0.1604****	−0.0026	0.5287****
CCA	0.0157****	0.0046*	**2.3180**	0.0759****	0.1871****
SE	0.0142****	0.004	0.0075****	**4.2944**	0.3122****
TA	0.0161****	0.0215****	0.0151****	0.0094***	**1.4330**

Significance levels denoted as follows:

**** <0.00001,

*** <0.001,

* <0.05.

**Table 4:** Measures of genetic diversity averaged across the number of samples and 12 microsatellite loci for each of the populations sampled in southern Kenya and northern Tanzania; A is the average number of alleles across all loci, H_E_ is the expected heterozygosity and H_O_ is the observed heterozygosity.

	A	H_E_	H_O_
AM	7.25	0.684	0.681
TA	5.75	0.669	0.655
CCA	7.25	0.666	0.656
MM	6.67	0.662	0.690
SE	6.67	0.660	0.660
Total	6.72	0.668	0.668

The elephants in the recently established population in the CCA had high levels of both mtDNA nucleotide and microsatellite allelic diversity when compared with the other populations (Tables 3 & 4). With six haplotypes detected, they had the second highest mtDNA nucleotide diversity, with two haplotypes (B & H) that were not detected in any individuals sampled in the park populations.

**Figure 2:**
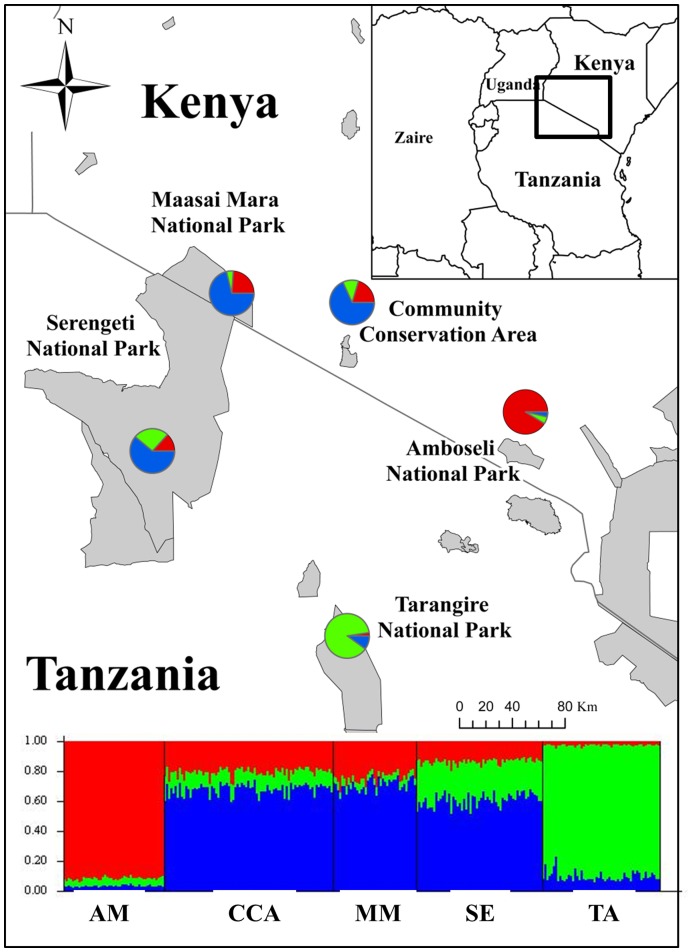
Individual Population Assignment from STRUCTURE. Barplot from STRUCTURE depicting population assignment for individuals mapped and sorted by sampling location: Amboseli (AM), Community Conservation Area (CCA), Maasai Mara (MM), Serengeti (SE) and Tarangire (TA); the Rift Valley splits the study area, Maasai Mara and Serengeti are on the west side of the valley, Amboseli and Tarangire are on the east side of the valley while the CCA is located within the Rift Valley.

The F_ST_ values for the nuclear markers were small relative to those based on mtDNA, but they indicated significant differentiation between all pairs of populations evaluated except Maasai Mara and Serengeti (Table 3). However, the elephants in the CCA showed the least genetic distance from the elephants sampled in the Maasai Mara, followed by Serengeti, with F_ST_ values an order of magnitude higher for Amboseli and Tarangire. The STRUCTURE analysis of the microsatellite data showed patterns similar to the F_ST_ analysis. From the five populations coded in the data using the LOCPRIOR option, the Evanno method [44] reported a value of K = 3 where the CCA, Maasai Mara and Serengeti elephants were all assigned to one population (Fig. 2; Figs. S1 & S2). To ensure the LOCPRIOR option was detecting actual population structure and not only geographic locations, we tested the method by coding Amboseli as the same population as Tarangire, and the results were the same, K = 3, with the same population assignments. The STRUCTURE models run without any information and under the POPFLAG option did not detect any population structure.

The genetic metapopulation pattern and evidence for a source population for the CCA elephants was stronger for the mtDNA data. All of the F_ST_ values yielded by the mtDNA data were much higher than for the microsatellite data but the patterns remained the same (Table 3). The only population pair that was not significantly differentiated was Maasai Mara and Serengeti, and the CCA elephants were found to be most similar to Maasai Mara and Serengeti. Because mtDNA F_ST_ values were large between parks, we split Serengeti, the largest park, into four distinct regions where collection took place (Fig. S3) and found the samples at the southern end of the park had a significantly different F_ST_ value from the other three Serengeti regions as well as Maasai Mara (Table 5). The three northernmost regions were not significantly different from Maasai Mara. Interestingly, the southern region was not significantly different from Tarangire.

**Table 5:** F_ST_ value comparison for sampled regions in Serengeti National Park, Tanzania based on mtDNA; F_ST_ values are below the diagonal and significance levels are above; sample names correspond to the northern (NSE), western (WSE), central (CSE) and southern (SSE) regions of Serengeti in addition to Maasai Mara (MM) in Kenya and Tarangire (TA) in Tanzania.

	MM	NSE	WSE	CSE	SSE	TA
MM	−				****	****
NSE	−0.02	−			*	****
WSE	−0.00	0.01	−		*	****
CSE	0.05	−0.02	0.11	−	***	****
SSE	0.28	0.18	0.18	0.22	−	0.05
TA	0.53	0.61	0.44	0.68	0.15	−

Significance levels denoted as follows:

**** <0.00001,

*** <0.001,

* <0.05.

Finally, the patterns of mtDNA haplotype distribution across the landscape were striking. The populations on the west side of the rift valley, Maasai Mara and Serengeti, showed very little haplotype overlap with the populations toward the east side of the rift valley, Amboseli and Tarangire (Fig. 1). The only haplotypes shared across the rift valley were from a few individuals sampled at the southern end of Serengeti National Park and a few individuals sampled at Tarangire. The landscape patterns also indicate Maasai Mara and Serengeti as the source populations for the elephants in the CCA. Although a few individuals in the CCA possessed one of the dominant haplotypes detected at Amboseli (haplotype A), all of them were adult males.

**Figure 3:**
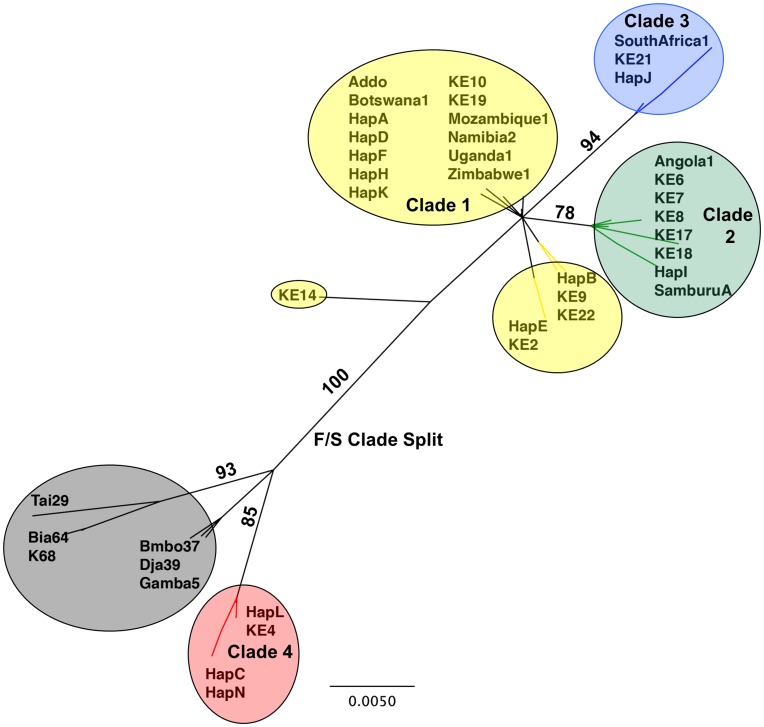
Neighbour-joining Tree for Savannah Elephant mtDNA Haplotypes. Unrooted neighbour-joinging tree using the HKY model in Geneious showing the relationship between 12 mtDNA haplotypes from elephants in Kenya and Tanzania and including published haplotypes in GenBank from Okello et al. [4], Eggert et al. [7] and Debruyne [17]; statistical support shown from 1000 bootstrap replicates. Clade nomenclature (Okello Clade 1–4) as denoted by Okello et al. [4] (yellow = Clade 1, green = Clade 2, blue = Clade 3, red = Clade 4, gray = Loxodonta cyclotis); F/S Split as denoted by Debruyne [17]. Hap A-F, H-L, and N represent the 12 haplotypes identified in this study.

The unrooted neighbour joining tree displayed similar structure to the phylogenetic tree of Kenyan haplotypes by Okello et al. [4] (Fig. 3). A similar grouping of four clades was indicated with the largest split between Okello’s clade four and the other three clades; this also corresponds to the F/S clade split described by Debruyne [17]. Most of the savannah elephant haplotypes added to the tree grouped with clades one through three, but clade four (including our haplotypes C, N and L in Fig. 1) grouped with haplotypes from forest elephants from west and central Africa (Fig. 2). Clade four is the dominant clade on the west side of the rift valley in both Kenya and Tanzania and rarely occurs on the east side of the rift valley [4] (Fig. 3).

## Discussion

Even though savannah elephants tend to have low nuclear genetic diversity, sometimes making it difficult to distinguish populations or locations [8], [26], [46], we were able to discern population structure in our study area using both mtDNA and nuclear DNA markers. We found a clear segregation of mtDNA haplotypes between the east and west sides of the rift valley in southern Kenya and northern Tanzania, and although the signal is not as strong, this separation was also detected with the nuclear DNA using both F_ST_ values and STRUCTURE. Similar patterns of genetic structure have been observed across the rift valley in other ungulate species [47], such as the wildebeest (*Connochaetes taurinus*) and the hartebeest (*Alcelaphus buselaphus*), as well as in amphibians [48] and plants [49], [50]. Historical habitat and population fragmentation likely caused these patterns, and strong female philopatry has maintained the patterns over time.

The difference in the magnitude of F_ST_ values observed between nuclear and mtDNA and sampling locations are informative about broad scale movement patterns for elephants in the region. The strong geographic patterns in the mtDNA sequences suggest high levels of female philopatry, while the weaker signal in the nuclear DNA suggests male-mediated gene flow from males mating outside natal herds [10], [14]. Similar patterns of genetic structure have been documented in other parts of East Africa [9]. The microsatellite F_ST_ differentiation that is observed among the sampling locations is likely driven by geographic isolation and could be somewhat attributable to sire effects [51]–[53]. However, population size is likely not a factor influencing the F_ST_ values. The Amboseli population is the smallest among the parks sampled, but the F_ST_ values between Tarangire and Maasai Mara and the CCA were as high or higher than the F_ST_ values between Amboseli and other locations. The mtDNA patterns were strongest across the northern end of the study area. We found no shared haplotypes between Maasai Mara and Amboseli, and only four elephants in the CCA, all of which were adult males, had a haplotype shared with Amboseli (haplotype A). Across the southern end of the study area, however, there was more evidence of haplotype mixing, suggesting more movement or connectivity in this part of the study area. This was also supported by the lower F_ST_ values for the nuclear microsatellites between Tarangire and Serengeti with the highest F_ST_ value was found between Tarangire and Maasai Mara. Serengeti had the highest haplotype diversity, and most of this haplotype diversity was detected at the southern end of the park. Serengeti also showed more haplotype overlap with elephants from Tarangire for both males and females, but this haplotype sharing was restricted to the southern region where there actually was no significant difference between the two parks, indicating that there may be movement of males and females between populations in those two parks.

The neighbour-joining tree denoted similar phylogenetic relationships of the mtDNA haplotypes to what has been previously reported [4], [7], but the geographic pattern of relationships in our study area is intriguing. We detected four well-differentiated clades similar to those found by Okello et al. [4]. When we added the additional published savannah and forest elephant haplotypes to the tree, we found that most of the savannah elephant haplotypes grouped in clades one, two and three, corresponding to Debryune’s S clade [17]. The forest elephant haplotypes clearly grouped with clade four, corresponding to Debryune’s F clade [17]. The prevalence of the F clade in these savannah elephants is likely explained by the cytonuclear dissociation patterns previously reported by Roca et al. [18]. However, it is interesting that clade four is geographically restricted. These haplotypes are by far the most common on the west side of the rift valley in Maasai Mara and Serengeti with rare occurrences on the east side of the valley in Tarangire. Given the strong dominance of clade four on the west side of the rift valley, it is possible that this area represents the eastern most extent of past forest habitat or one of the Pleistocene refugia for rainforest and therefore forest elephants, allowing introgression between forest and savannah elephants [13], [53]. The prevalence of clade four in Maasai Mara and Serengeti likely also corresponds to the predominant east-central F subclade reported in Serengti by Ishida et al. [13]. The fact that the F clade is dominant on the west side of the Rift Valley and nearly absent from the east side of the valley is likely reinforced by the strong philopatry of female elephants. This pattern, however, is very useful for helping to determine the origin of the elephants that have recolonized the CCA in the Rift Valley.

Our mtDNA and nuclear data clearly demonstrate that the elephants that recolonized the CCA came from the west side of the rift valley (Maasai Mara and Serengeti). While movement outside of Maasai Mara and Serengeti has been demonstrated, particularly in crop-raiding situations [54], this is the first documentation of a new dispersal and recolonization event on community-owned land for the region. The elephants in this area have become resident; many of the same males and females were detected in the area across years [55]. This suggests that the growing elephant population in Serengeti and Maasai Mara is resulting in movement into areas far from the borders of the parks. It also suggests that elephants can independently colonize Community Conservation Areas in highly human-dominated landscapes where pastoral land uses are compatible.

The trans rift valley area along the Kenya and Tanzania border is one of the remaining strongholds for savannah elephants, supporting many fragmented populations [5]. However, most of the parks in the region are not large enough to maintain viable populations of large herbivores [56], including elephants [19]. Therefore, maintaining a viable population in the region is likely to require a metapopulation approach, which will involve strategic protection of habitat and corridors outside the boundaries of the established protected areas to connect the currently isolated populations. Our data suggest that some population mixing may currently be occurring at the southern end of the study area, and the recent colonization event in the CCA suggests that populations on the west side of the rift valley are already expanding. The independent colonization of the CCA by the elephants suggests that a strategically designed network of CCAs across the human-dominated landscape between the parks could be successful at allowing elephants to independently colonize these areas and serve as stepping-stones between the larger populations, which would help maintain genetic exchange between elephants living in protected areas in the rift valley. These landscape designs and conservation plans are urgently needed, as human populations and habitat fragmentation continue to grow in the region [57], rapidly decreasing the potential for keeping the landscape open for elephants and other wildlife.

Genetic evidence has helped identify movement pathways across the rift and into human-dominated areas. Protection of these expanding populations can help ensure population persistence inside the protected areas, and will necessitate cross-border collaboration and further research to identify and protect additional corridors and suitable habitat. Establishment and deployment of a network of national and local community scouts may also be necessary to protect these important populations of elephants as they expand their range to colonize new areas.

## Supporting Information

Figure S1 Barplots from STRUCTURE using LOCPRIOR depicting population assignment for individuals mapped and sorted by sampling location for K = 2 though K = 5: Amboseli (AM), Community Conservation Area (CCA), Maasai Mara (MM), Serengeti (SE) and Tarangire (TA); the Rift Valley splits the study area, Maasai Mara and Serengeti are on the west side of the valley, Amboseli and Tarangire are on the east side of the valley while the CCA is located within the Rift Valley.
(TIF)

Figure S2 Plot of Delta K from STRUCTURE outputs run from K = 1 to K = 9 using locprior option; output obtained from STRUCTURE Harvester.
(TIF)

Figure S3 Map of the sample locations and groupings in Serengeti National Park, Tanzania for testing within park F_ST_ structure using mtDNA haplotypes; names correspond to the southern (SSE), central (CSE), western (WSE) and northern (NSE) groupings.
(TIF)

Table S1 Number of each haplotype detected in each of the five sampled populations in southern Kenya and northern Tanzania in 2007 and 2008.
(DOCX)

Table S2 Allele size ranges in bp for each of 12 loci across five populations in southern Kenya and northern Tanzania; all loci except those marked are dinucleotide repeats.
(DOCX)
