# Supplementary figures and images for: Identifying Source Populations and Genetic Structure for Savannah Elephants in Human-Dominated Landscapes and Protected Areas in the Kenya-Tanzania Borderlands
Source: PLoS One. 2012 Dec 26;7(12):e52288. doi: 10.1371/journal.pone.0052288 (PMC3530563; doi:10.1371/journal.pone.0052288)

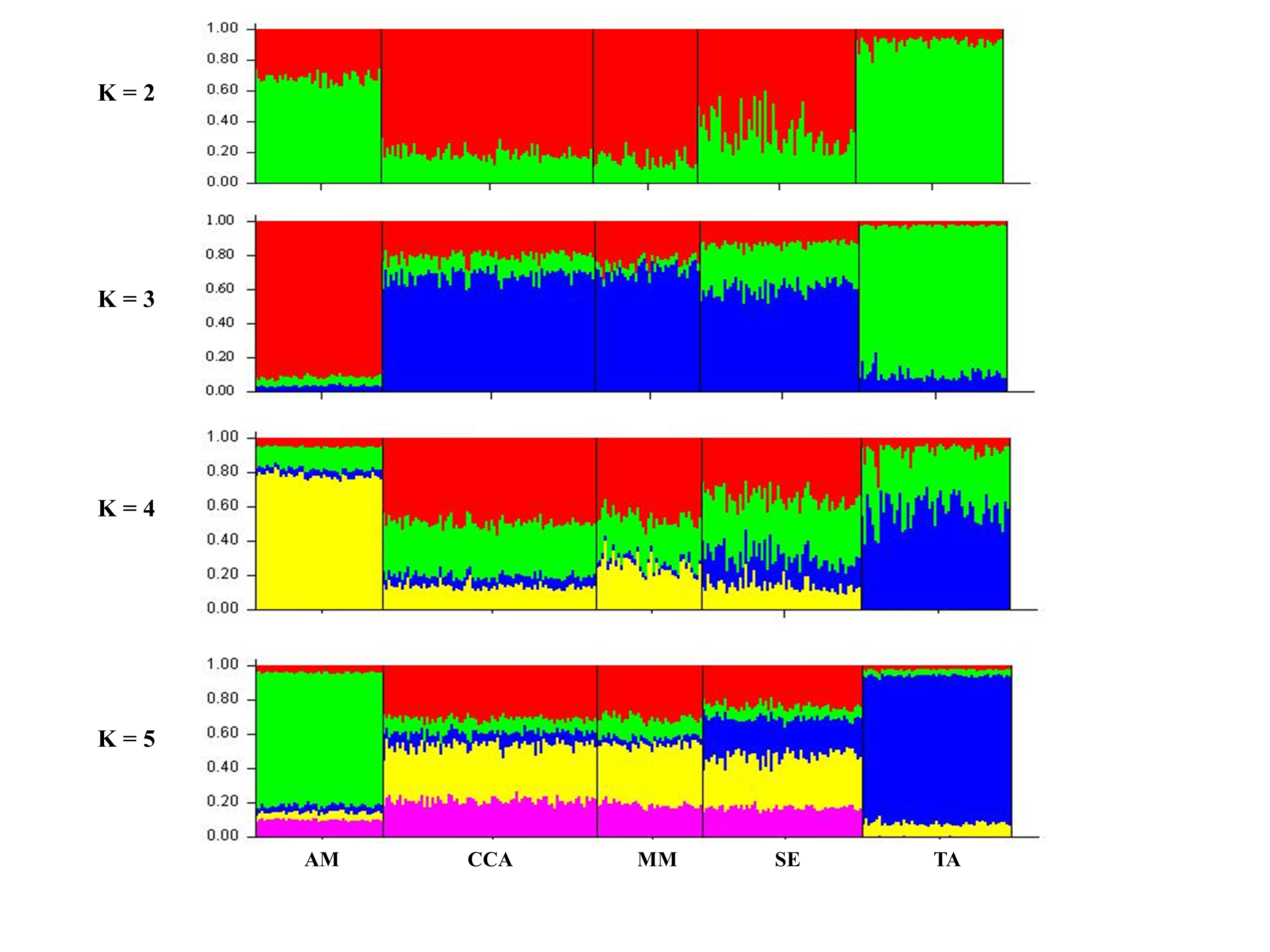

Supplement: Figure S1 — Barplots from STRUCTURE using LOCPRIOR depicting population assignment for individuals mapped and sorted by sampling location for K = 2 though K = 5: Amboseli (AM), Community Conservation Area (CCA), Maasai Mara (MM), Serengeti (SE) and Tarangire (TA); the Rift Valley splits the study area, Maasai Mara and Serengeti are on the west side of the valley, Amboseli and Tarangire are on the east side of the valley while the CCA is located within the Rift Valley. (TIF) [file pone.0052288.s001.tif]

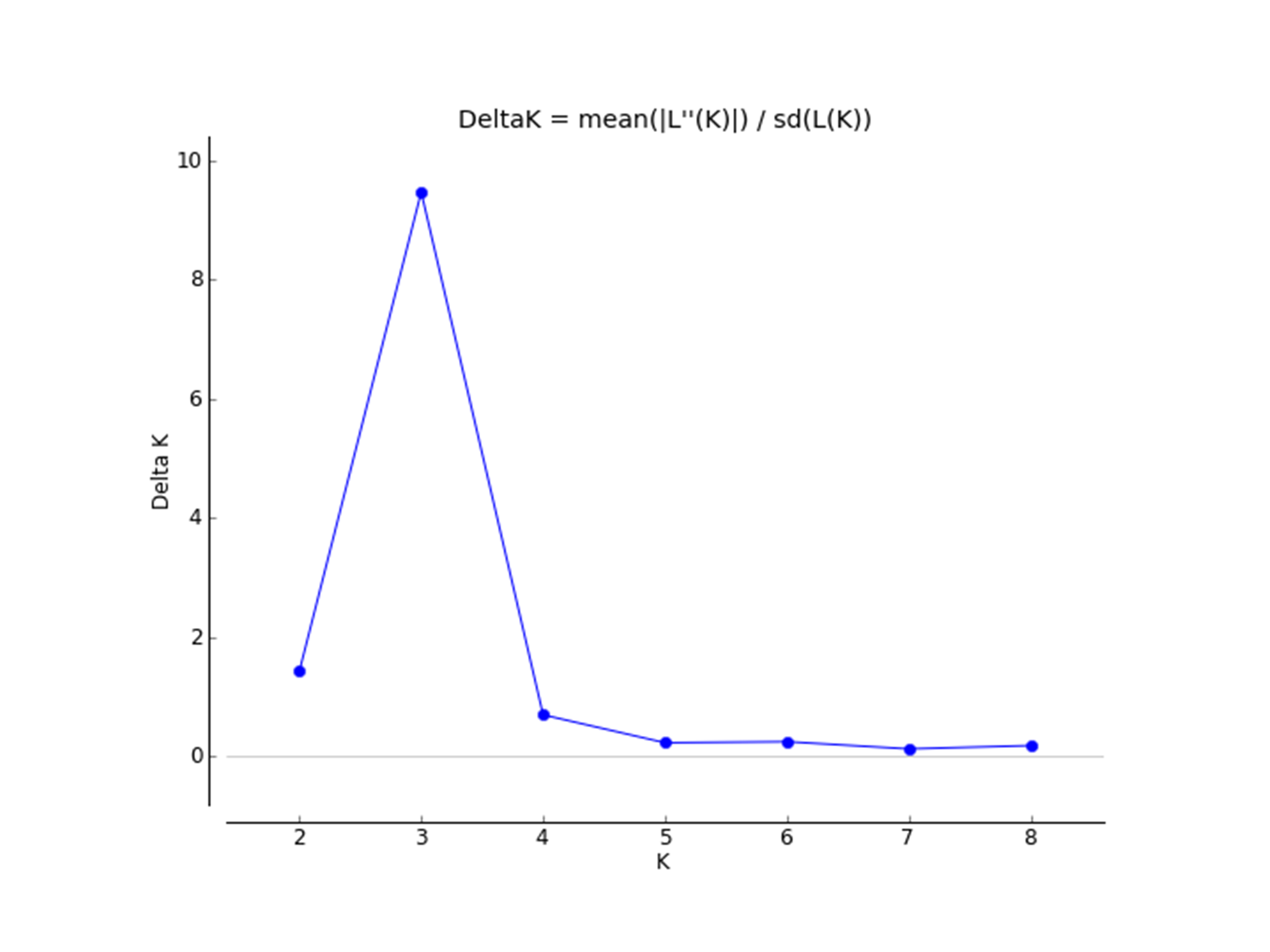

Supplement: Figure S2 — Plot of Delta K from STRUCTURE outputs run from K = 1 to K = 9 using locprior option; output obtained from STRUCTURE Harvester. (TIF) [file pone.0052288.s002.tif]

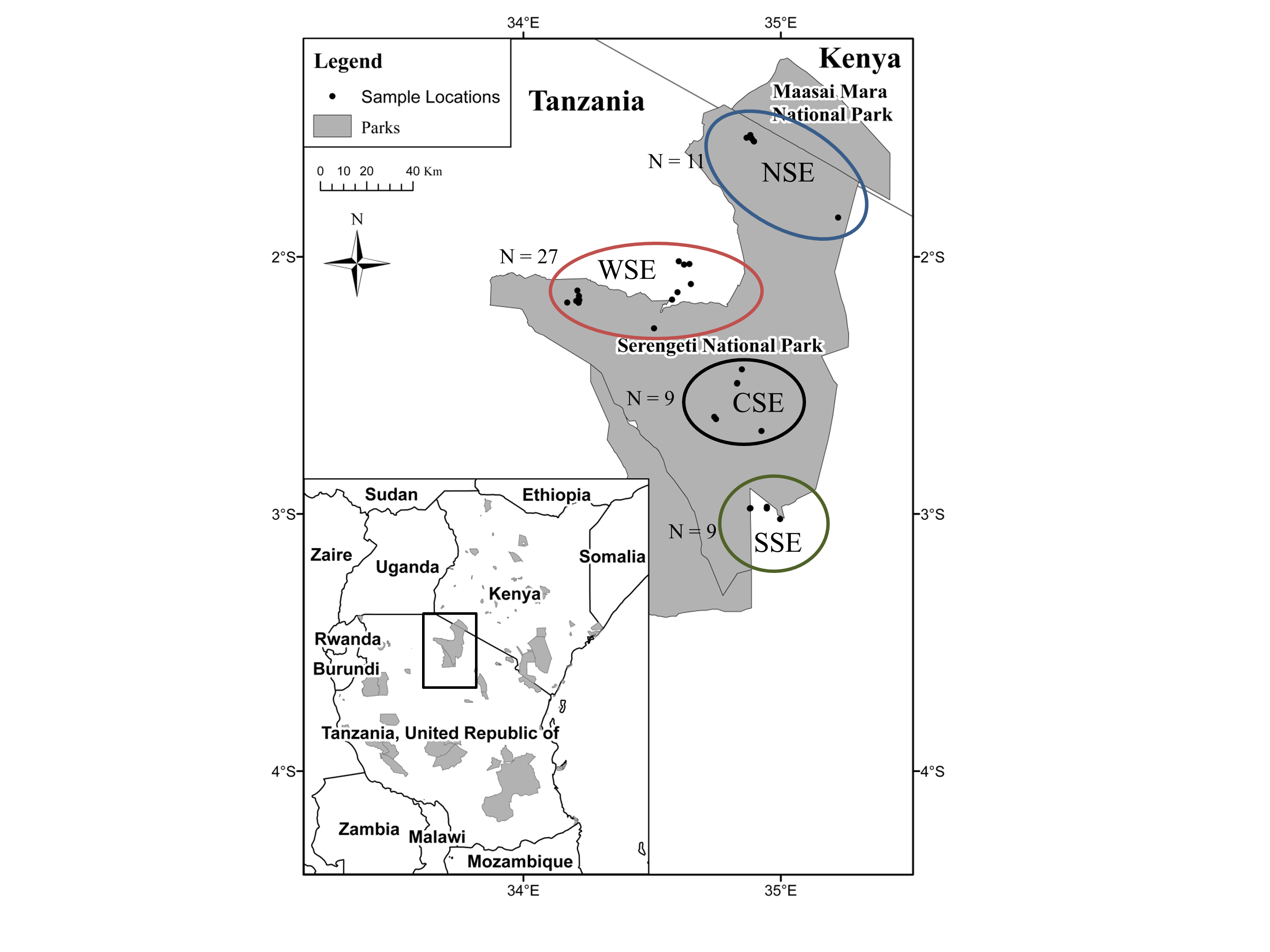

Supplement: Figure S3 — Map of the sample locations and groupings in Serengeti National Park, Tanzania for testing within park FST structure using mtDNA haplotypes; names correspond to the southern (SSE), central (CSE), western (WSE) and northern (NSE) groupings. (TIF) [file pone.0052288.s003.tif]
