# Supplementary material for: Identifying Source Populations and Genetic Structure for Savannah Elephants in Human-Dominated Landscapes and Protected Areas in the Kenya-Tanzania Borderlands
Source: PLoS One. 2012 Dec 26;7(12):e52288. doi: 10.1371/journal.pone.0052288 (PMC3530563; doi:10.1371/journal.pone.0052288)
Supplement: Table S1 — Number of each haplotype detected in each of the five sampled populations in southern Kenya and northern Tanzania in 2007 and 2008. (DOCX) [file pone.0052288.s004.docx]

Table S1. Number of each haplotype detected in each of the five sampled populations in southern Kenya and northern Tanzania in 2007 and 2008.

| **Population** | **A** | **B** | **C** | **D** | **E** | **F** | **H** | **I** | **J** | **K** | **L** | **N** |
| --- | --- | --- | --- | --- | --- | --- | --- | --- | --- | --- | --- | --- |
| Amboseli | 18 | 0 | 0 | 0 | 22 | 7 | 0 | 0 | 0 | 0 | 0 | 0 |
| Maasai Mara | 0 | 0 | 32 | 1 | 0 | 0 | 0 | 6 | 0 | 0 | 0 | 0 |
| Community Conservation Area | 4 | 6 | 58 | 38 | 0 | 0 | 4 | 2 | 0 | 0 | 0 | 0 |
| Serengeti | 5 | 0 | 38 | 6 | 0 | 0 | 0 | 1 | 1 | 1 | 4 | 0 |
| Tarangire | 37 | 0 | 2 | 0 | 0 | 7 | 0 | 0 | 1 | 0 | 0 | 8 |
