# Supplementary material for: Identifying Source Populations and Genetic Structure for Savannah Elephants in Human-Dominated Landscapes and Protected Areas in the Kenya-Tanzania Borderlands
Source: PLoS One. 2012 Dec 26;7(12):e52288. doi: 10.1371/journal.pone.0052288 (PMC3530563; doi:10.1371/journal.pone.0052288)
Supplement: Table S2 — Allele size ranges in bp for each of 12 loci across five populations in southern Kenya and northern Tanzania; all loci except those marked are dinucleotide repeats. (DOCX) [file pone.0052288.s005.docx]

Table S2. Allele size ranges in bp for each of 12 loci across five populations in southern Kenya and northern Tanzania; all loci except those marked are dinucleotide repeats.

| **Population** | **FH60R** | **FH48R** | **LA4** | **FH94R** | **LA6R** | **LA5** | **FH67** | **FH126** | **LaT05*** | **LaT13R*** | **LaT08*** | **LafMS02** |
| --- | --- | --- | --- | --- | --- | --- | --- | --- | --- | --- | --- | --- |
| Amboseli | 6 | 14 | 6 | 6 | 10 | 8 | 14 | 26 | 52 | 36 | 60 | 12 |
| Maasai Mara | 6 | 12 | 6 | 8 | 8 | 7 | 18 | 20 | 60 | 28 | 40 | 10 |
| CCA** | 6 | 14 | 6 | 8 | 18 | 5 | 14 | 20 | 72 | 40 | 52 | 12 |
| Serengeti | 6 | 16 | 6 | 8 | 18 | 5 | 14 | 20 | 68 | 32 | 36 | 12 |
| Tarangire | 6 | 10 | 6 | 6 | 8 | 4 | 24 | 18 | 28 | 36 | 28 | 12 |

*Loci are tetranucleotide repeats

**Community Conservation Area
